# Analysis of Temperature Influence on Strain–Speed Parameters of Radial-Shear Rolling of Al-Zn-Mg-Ni-Fe Alloy

**DOI:** 10.3390/ma15207202

**Published:** 2022-10-15

**Authors:** Sergei P. Galkin, Yury V. Gamin, Tatiana Yu. Kin

**Affiliations:** Department of Metal Forming, National University of Science and Technology «MISIS» (NUST MISIS), 4 Leninsky pr., Moscow 119049, Russia

**Keywords:** aluminum alloy, Al-Zn-Mg-Ni-Fe, contact slippage, equivalent strain, length of trajectory, radial-shear rolling, strain rate intensity, temperature conditions

## Abstract

The comprehensive analysis of temperature influence on the strain–speed parameters of radial-shear rolling of Al-Zn-Mg-Ni-Fe alloy including the investigation of rheological properties, FEM simulation, and in-depth analytical interpretation of results was carried out. The rolling temperature has significant effect on the kinematic of metal forming, speed parameters, configuration, and length of trajectories. With the decrease in temperature, the speed of metal movement reduces, and this is the same for different components. The greatest decrease is noted for the axial speed component. In general, according to the nature of effect on the strain kinematic state, a temperature reduction of 100 °C (from 500 to 400 °C) acts similarly to a decrease in feed angle of about 4° and, in particular, increases the rolling time, nonuniformity of deformation, tightening, and temperature effect of deformation heating.

## 1. Introduction

High-strength aluminum alloys are ones of the widely used structural materials in the aviation, missile, and automobile industries as well as and other technical fields [1,2,3,4]. This group of alloys includes alloys of the Al-Zn-Mg-Cu system of the 7xxx series. With the development of technologies and technical systems, there is a natural increase in the requirements of the properties of materials. For these reasons, such high-strength alloys as V96ts, V96pch, etc., were invented [5,6,7]. The economically alloyed nikalin aluminum alloys based on the Al–Zn–Mg–Fe–Ni system were developed at NUST “MISIS” [8,9,10]. The studies have shown [8,11,12] that the mechanical properties of nikalins of the AC6N0.5Zh type (Al—(6.0–7.0)% Zn—(2.0–3.0)% Mg—(0–0.2)% Cu—(0.4–0.7)% Fe—(0.5–0.8)% Ni—(0–0.2)% Zr) are at the level of known high-strength alloys of the V95/V96 type and in some respects even surpass them. High-strength characteristics in combination with good ductility in the new alloy are achieved due to the uniform distribution over the aluminum matrix of precipitates after aging of nanosized dispersoids, as well as fine particles of the Al_9_FeNi phase. The different semi-finished products, such as sheets, bars, stampings, and wire, were made from nikalin AC7NZh, which indicated the possibility of its use as deformed semi-finished products [11,13,14,15,16].

The previous studies carried out in NUST «MISIS» shown [13,15] that process of radial-shear rolling (RSR) was effective method of alloy hardening. Due to severe shear strains in the surface layers of the workpiece, the process of microstructure refinement occurs similar to the processes of severe plastic deformation (SPD) [17]. However, the detailed analysis of influence of temperature and strain-speed modes of RSR was not carried out. In the article [18] the comparative study of RSR conditions for 7075 and Al7Zn2.8Mg0.7Ni0.55Fe0.2Zr alloys was shown. The influence of high-temperature RSR on the distribution of stresses and strains over cross section of bars was studied. The data obtained show that Al7Zn2.8Mg0.7Ni0.55Fe0.2Zr prototype alloy had a greater tendency to develop shear strains and show their localization on the bar surface. At the same time, the reasons for this behavior of material were not discussed.

Full-scale experimental studies do not allow the practical analysis of the process parameters specific to RSR, such as the trajectories of metal flow in the deformation zone, the distribution of slippage on contact depending on temperature, etc. With the development of computer programs for simulation, the use of the finite element method (FEM) is an available solution to this problem. Software systems for the simulation of metal-forming processes allow engineering analysis of the main process parameters to be performed without significant time and financial costs [19,20,21,22,23,24].

The main objective of this article is analysis of influence of rolling temperature on the strain–speed parameters of RSR of Al-Zn-Mg-Ni-Fe alloy.

## 2. Materials and Research Methods

The analysis was carried out based on the results obtained using the Qform 3D software package designed for computer FEM simulation and the computational and analytical interpretation. The simulation was performed for an aluminum alloy of Al-Zn-Mg-Ni-Fe system, its chemical composition corresponded to the AZ6NF alloy according to GOST 4784-2019.

**Figure 1:**
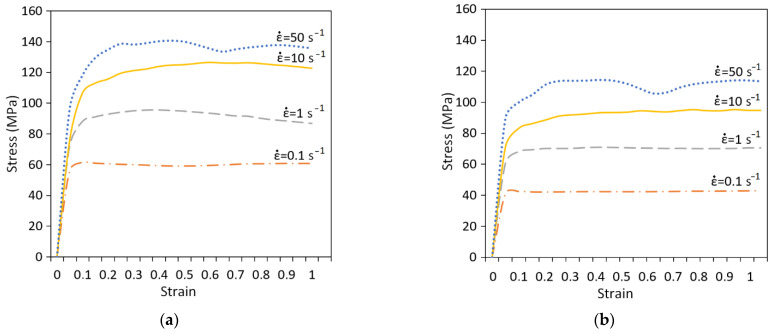

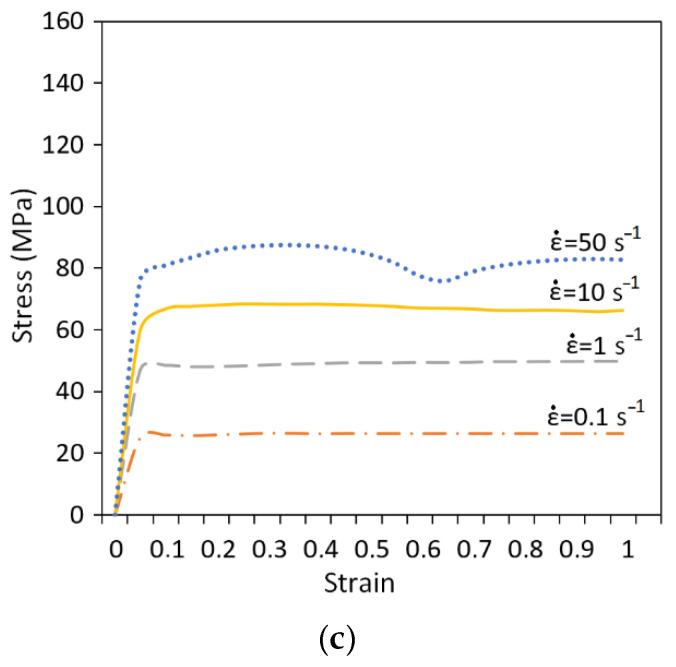
Resistance to deformation of Al-Zn-Mg-Ni-Fe alloy at deformation temperature: (**a**) 400 °C; (**b**) 450 °C; (**c**) 500 °C.

The calculation of parameters in the Qform is based on the model of material which is described with rheological and thermophysical properties. The resistance to deformation *σ* is a function of temperature *T*, strain rate $\dot{\varepsilon }$, and strain *ε*. The data on the resistance to deformation of investigated alloy were obtained as a result of compression tests using the Gleeble 3800 thermal–mechanical physical simulation system at different temperatures and strain rates (Figure 1).

**Figure 2:**
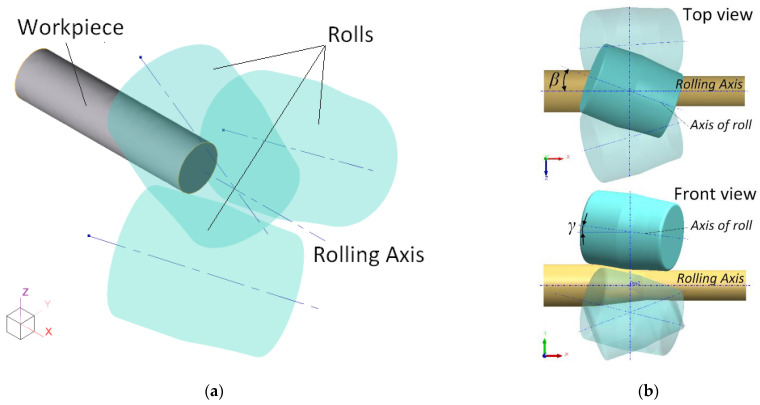
3D model for simulation (**a**) and scheme of roll angles for the RSR process (**b**).

The designed 3D model consists of three rolls and a workpiece (Figure 2). The initial workpiece axis coincides with the rolling axis; each roll is turned through the feed angle *β* = 20° and toe angle *γ* = 7°. The distance from the pitch point of each roll to the rolling axis is set equal to the bar’s radius.

The main parameters given in the pre-processor program are presented in Table 1.

According to the results of simulation, the temperature distribution of metal in the deformation zone, strain, speed, and force parameters were evaluated.

The equivalent strain in the QForm is calculated by numerically integrating the strain rates intensity for each node using the formula:

(1) $$\varepsilon =\underset{t}{\sum }\dot{\varepsilon }\Delta t_{n},$$

where $\Delta t_{n}$ is a time step of calculation.

The trajectory–speed parameters, such as angles and length of helical trajectories, metal flow speeds, and their axial and circumferential components are among the most important characteristics of radial-shear rolling. The information on these parameters cannot be explicitly obtained with the QForm program interface. For this reason, the in-depth computational and analytical interpretation of the simulation results is required based on the theoretical parts of studies [25,26] using the formulas given below.

**Figure 3:**
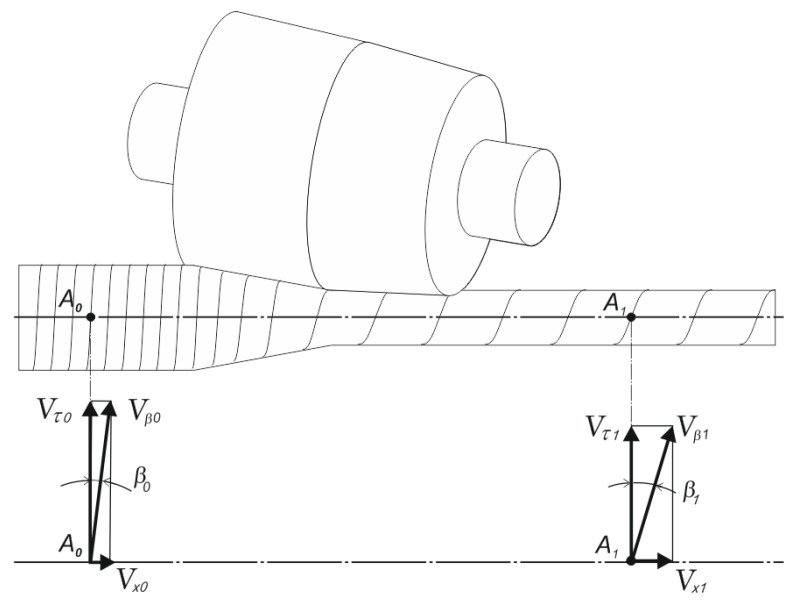
The trajectory–speed scheme of metal flow during the RSR.

During the RSR, the metal flow in deformation zone occurs along the helicoidal trajectories of different lengths (Figure 3), and it has a cyclic character. In the three-roll mill, one cycle of deformation corresponds to revolution of the workpiece by 1/3 of a turn—120°.

In the deformation zone, the speeds of metal flow, their components, and parameters of helicoidal trajectories are changed.

In the stationary process of RSR, the trajectories of movement coincide with the lines of flow. Therefore, the equation for determining the angles of helical trajectory before the reduction by rolls *β_0_* and after the reduction by rolls *β*_1_ (Figure 3) is as follows:

(2) $$tan\;\beta_{1\left(0\right)}=\frac{V_{x1\left(0\right)}}{V_{\tau 1\left(0\right)}},$$

where $V_{x1\left(0\right)},V_{\tau 1\left(0\right)}$ are the axial and circumferential components of the metal speed on the rolled surface, respectively. The index 0 means “before deformation zone”, and the index 1 means “after deformation zone”.

It is important to note that the angles of trajectories are the main factors of controlling the screw rolling process, which largely determines the result of deformation [26].

The deformation zone consists of two main sections—a reduction zone in the shape of a truncated cone and a calibration zone that has a shape close to that of a cylinder. The trajectories of points lying on the surface of workpiece have the greatest length *L_β_*, while those lying on the rolling axis have the shortest length *L_oc_.* The length of axial trajectory in reduction zone with cone angle *α* coincides with the length of this zone and can be determined as follows:

(3) $$L_{oc}=\frac{d_{1}\cdot \left(\sqrt{\mu }-1\right)}{2\cdot tan\;\alpha },$$

where *d*_1_ is a diameter of bar; *μ* is elongation ratio.

The number of deformation cycles *N* in the reduction zone is determined through the volume constancy law as the ratio of the volume of a truncated cone to the volume of metal coming out of rolls after 1/3 of the workpiece revolution. The larger cone base of reduction zone has the diameter of initial workpiece $\sqrt{\mu }\cdot d_{1}$, and the smaller one equals to bar diameter:

(4) $$N=\frac{1}{C}\left(\mu^{\frac{3}{2}}-1\right)$$

where *C* is a constant of the deformation zone and equals:

(5) $$C=2\pi \cdot tan\;\beta_{1}\cdot tan\;\alpha .$$

The length of the surface trajectory *L_β_* in the deformation zone can be approximately calculated based on the volume constancy law taking into account the elongation ratio *μ*, the angle of trajectory *β*_1_, and the number of deformation cycles *N* [24,25]:

(6) $$L_{\beta }=\frac{\pi \cdot d_{1}\cdot N\cdot {\left(tan^{2}\beta_{1}+\mu^{3}\right)}^{\frac{1}{2N}}}{3\cdot \mu^{\frac{1}{N}}\cdot {\left(tan^{2}\beta_{1}+1\right)}^{\frac{1}{2}\left(\frac{1}{N}-1\right)}}.$$

The relative error of Equation (6) does not exceed 10%.

In the QForm, the length of trajectories is calculated using the tracking points on the surface and on the axis of the workpiece. The length increment of trajectory Δ*L_i_* at the *i*-th step is determined by the change in the point coordinates $\Delta X_{i,}\;\Delta Y_{i\;,}\Delta Z_{i,}$ relative to the previous position:

(7) $$\Delta L_{i}=\sqrt{\Delta X_{i}{}^{2}+\Delta Y_{i}{}^{2}+\Delta Z_{i}{}^{2}}.$$

The sum of all $\Delta L_{i}$ on the length of deformation zone for each of the tracking points will be the required lengths of trajectories for the axial zone *L_oc_* and the surface of bar *L_β_*.

## 3. Results and Discussion

Computational and analytical interpretation of the simulation results for the speed and trajectories of the metal flow is presented in Table 2.

According to the data obtained, the rolling temperature exerts significant influence on kinematical picture of metal forming and on the speed parameters, configuration, and length trajectories. With the decrease in temperature, the speed of metal movement reduces, and it is not the same for different components. The greatest decrease is observed for the axial component of speed. In particular, the decrease of *V*_x1_ on the exit from rolls is 33.4%. The tangential component of speed *V*_τ1_ decreases significantly less, by 11.3%, and total speed *V_β_*_1_ reduces by 14.2%.

**Figure 4:**
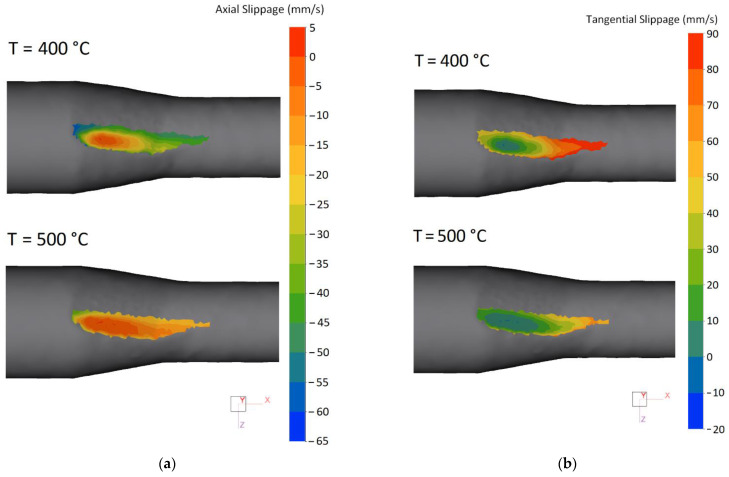
The field of (**a**) axial slippage and (**b**) tangential slippage on surface of workpiece contact with roll during RSR.

These changes are due to the temperature’s influence on the metal’s slippage relative to the surface of the rolls in the zone of contact interaction [27] that is shown in Figure 4. The axial slippage is the axial component of metal flow speed relative to the roll’s surface. It is calculated in QForm 3D for each node *M* on the contact surface as the difference between the axial component of the metal flow speed *V_x_*(*M*) and the axial component of the circumferential speed of rolls *U_x_*(*M*), i.e.,

(8) $$\Delta V_{x}\left(M\right)=V_{x}\left(M\right)-U_{x}\left(M\right).$$

The result is presented as the distribution of fields Δ*V_x_*(*M*) over the area of the contact surface (Figure 4a). The negative values of slippage Δ*V_x_*(*M*) mean the lagging of metal in the axial direction relative to the surface of the rolls, i.e, *V_x_*(*M*) < *U_x_*(*M*).

For the temperature of 500 °C the axial slippage Δ*V_x_*(*M*) is in the range from 5 to −20 mm/s on almost the entire contact surface (red–orange–yellow zone). With the decrease in rolling temperature to *T* = 400 °C, the axial slippage increases significantly, by 3 or more. On the contact surface in the zone of metal capture by rolls and in the zone of exit from rolls, light green–blue areas appear for levels of axial slippage from −30 to −60 mm/s.

The tangential slippage at point *M* of contact surface is determined as follows:

(9) $$\Delta V_{\tau }\left(M\right)=V_{\tau }\left(M\right)-U_{\tau }\left(M\right),$$

where *V*_τ_(*M*) is circumferential component of metal movement speed; *U*_τ_(*M*) is circumferential component of rotary velocity of rolls.

On almost the entire contact surface, the values of tangential slippage are in the neutral and positive zone, which corresponds to forward flow (Figure 4b). The range of change in Δ*V*_τ_(*M*) is 0–70 mm/s (green–orange zone) for a temperature of 500 °C and slightly expands to 0–90 mm/s (green-red zone) at the lower temperature of 400 °C. This shows that tangential slippage is less affected by temperature than axial slippage.

The rolling temperature exerts significant influence on configuration and length of trajectories of metal flow. As a result of the contact slippage, the deviation of direction of metal movement at the exit from deformation zone occurs and is characterized by the angle of helical trajectory *β*_1_ from the direction of circumferential speed vector of rolls, determined by the feed angle *β =* 20°. At the rolling temperature of 500 °C, *β*_1_ > *β*, and at 400 °C, *β*_1_ < *β*. This shows that the trajectories of metal movement deviate in different directions from the circumferential speed of rolls. In the first case, the trajectory deviates towards the direction of rotation by 2.6°, increasing the value of *β*_1_ to 22.6°. In the second case, the value increases by almost the same value up to the value of *β*_1_ = 17.4°. The angle *β*_1_ is one of the most significant factors in the RSR process. With its decrease, such parameters as the number of deformation cycles, the length of trajectories that are not on the workpiece axis, and the nonuniformity of deformation increase.

**Figure 5:**
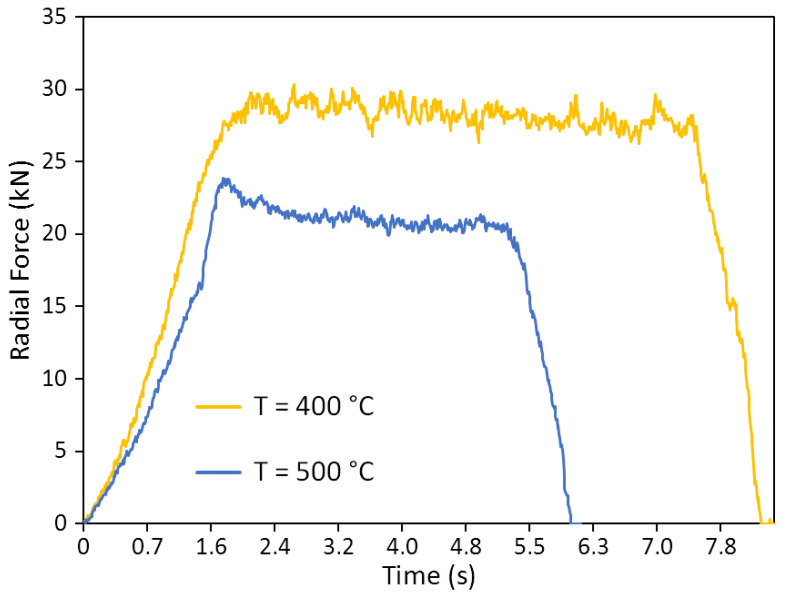
The diagram of change in the radial force on roll at different rolling temperatures.

The graphs of changes in the radial force (i.e., the force that acts on the roll from the workpiece during deformation and is directed perpendicular to the axis of the roll) depending on the process time are shown in Figure 5. Each graph is characterized by three stages of the process, such as:

- The stage of capture of metal by rolls, determined by the growth of radial force from 0 to the stationary level;
- The stationary stage, characterized by an almost horizontal section;
- The stage of metal exit from the deformation zone, at which the force of the metal on the roll decreases to 0.

The decrease in rolling temperature in the investigated range increases the radial force on roll from approximately 22 to 28 kN, i.e., by 15–20%. At the same time, the graphs record the increase in the length of horizontal section, i.e., the increase in the duration of stationary stage of process of almost 1.5 times (Figure 5). The increase in force is mainly due to the growth of resistance to deformation. The increase in the rolling time is directly related to the slowdown of the process, the decrease in the angle *β_1,_* and the axial component of *V*_x1_.

**Figure 6:**
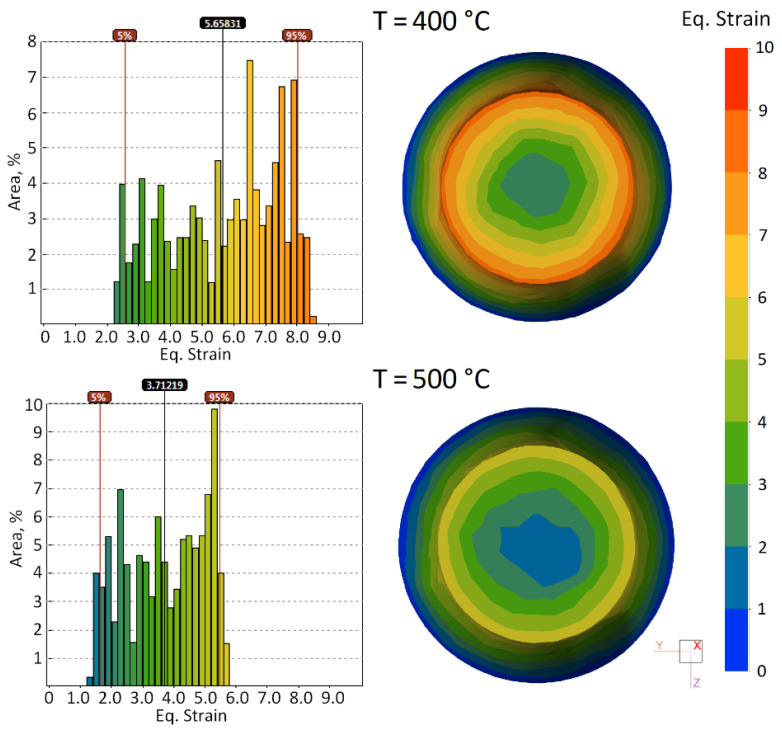
The field of equivalent strain distribution in cross section of the bar for different temperatures.

**Figure 7:**
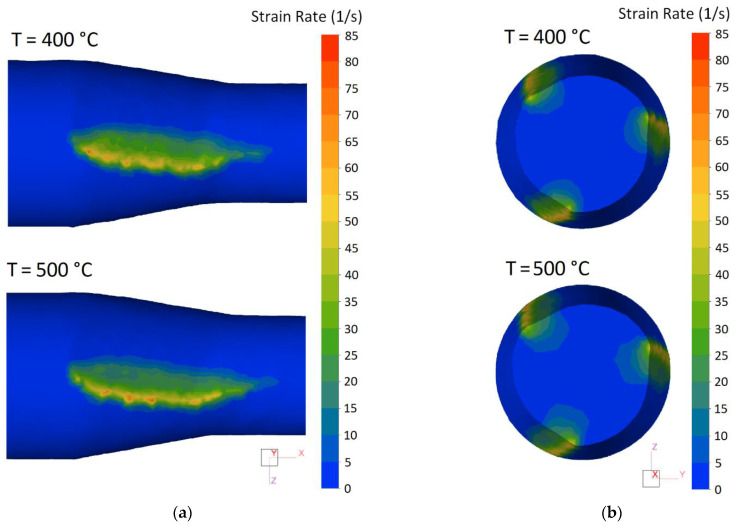
The field of strain rate distribution (**a**) on the surface of the workpiece in contact with roll and (**b**) in the cross section of the pitch point of the deformation zone.

The results of simulation are shown as the fields of equivalent strain *ε* in Figure 6 and as distribution of strain rate intensity $\dot{\varepsilon }$ in Figure 7. The general picture of fields obtained is marked by a nonuniformity (gradient) characteristic of radial-shear rolling with maximum values in the peripheral layers and minimal values in the central zone of workpiece.

From comparison of the data obtained, it follows that field of equivalent strain significantly depends on the rolling temperature, while the strain rate is related to temperature much more weakly.

It is known [28] that strain rate at screw rolling is determined by circumferential and angular speed of rotation of the deformed workpiece, i.e., by the parameters that vary within no more than 10–15% (Table 2 and Figure 7b) under conditions of this study.

It follows that the influence of rolling temperature on the maximum and median values of equivalent strain is almost completely due to the changes in the angle of surface trajectory *β*_1_ and axial speed of rolling *V*_x1_. With the decrease in these values, the time in the deformation zone of each elementary volume increases, i.e., the time interval that is used for integration in the calculation of equivalent strain directly increases. It is characteristic that maximum values of equivalent strain increase approximately from 5.2 to 8.0, i.e., by 1.53 times, with the decrease in the rolling temperature from 500 to 400 °C. Median values also are increased by 1.52 times from 3.71 to 5.65, i.e., this growth is almost as much as that of the rolling time shown above.

The fields in Figure 6 also can be explained in the alternative way. In stationary rolling process, when the flow trajectories do not depend on time, the equivalent strain for arbitrary point can be calculated by summing (integrating) along its trajectories over length *L*. For this, in Formula (1) the obvious replacement of the small step of calculation of *Δt_n_* is made with respect to time per step of calculation along the length of trajectory *ΔL_n_*.

(10) $$\Delta t_{n}=\frac{\Delta L_{n}}{V_{n}};$$

Next, the formula for *ε* is:

(11) $$\varepsilon =\underset{L}{\sum }\dot{\varepsilon }\frac{\Delta L_{n}}{V_{\beta n}},$$

where *V_βn_* is the movement speed of the considered node.

It directly follows that the increase in equivalent strain in the peripheral layers of the workpiece with the decrease in temperature is determined by the simultaneous stretching of trajectories and the reduction of movement speed of metal particles since the strain rate intensity insignificantly depends on the rolling temperature (Figure 7).

It can be seen from Table 2 that the length of trajectories of points on the workpiece axis does not depend on the rolling temperature and is approximately equal to the length of the deformation zone. On the contrary, the length of trajectory of points on the surface depends significantly on temperature, and it is approximately 30% longer for *T* = 400 °C than for 500 °C. In addition, with the decrease in temperature, the difference between the lengths of surface and axial trajectories increases. This leads to the increase in the gradient (difference) of equivalent strain over the workpiece section.

**Figure 8:**
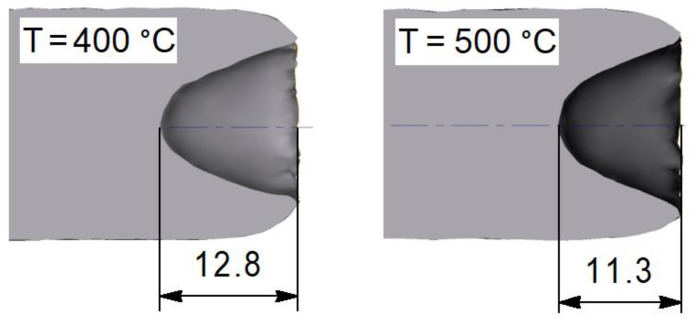
The depth of end-tightening of bars after RSR for different temperatures.

Obviously, the resulting difference in trajectories should be reflected in the deformation nonuniformity of bars after rolling. This nonuniformity can be evaluated by the size and shape of the tightening formed on the ends of bar. It can be seen from Figure 8 that tightening of bar rolled at lower temperature has more elongated shape in longitudinal section and greater depth ~12.8 mm.

**Figure 9:**
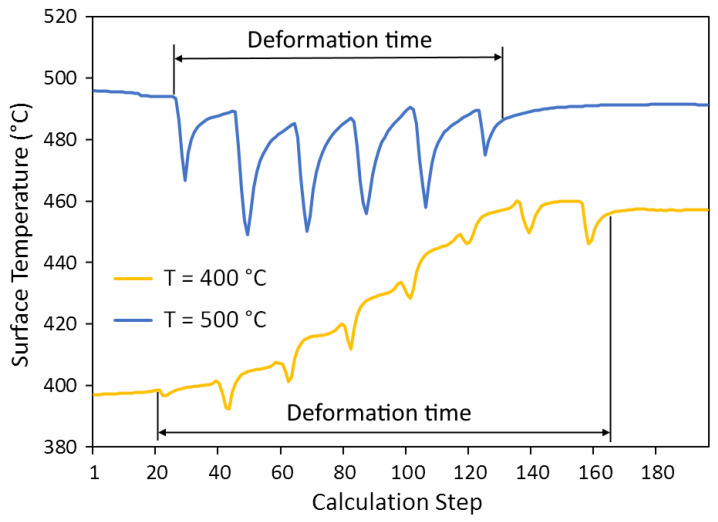
Change in temperature of point on bar surface along the deformation zone.

The temperature conditions in the deformation zone are formed under the action of two differently directed processes, such as deformation heating and natural heat transfer from the workpiece to the environment. The graphs of changes in temperature of bar surface as it passes through the deformation zone (calculation step) are shown in Figure 9. As it is known, the temperature effect of deformation heating is proportional to the strain rate and resistance to deformation. The higher resistance to deformation at *T* = 400 °C causes an increase in the surface temperature of rolled products of approximately 60 °C. At the rolling temperature of 500 °C and with lower resistance to deformation, the heating does not fully compensate for the natural heat loss and the rolled surface slightly cools (by about 10 °C).

Local minimums on the temperature curves are due to the contact of the tracked surface element with relatively cold rolls. Their number corresponds to the number of deformation cycles. It can be seen that with the decrease in temperature, the number of cycles increases because of the angle of surface trajectory *β*_1_ decreases.

## 4. Conclusions

Comprehensive analysis of temperature’s influence on the strain–speed parameters of radial-shear rolling of Al-Zn-Mg-Ni-Fe alloy including the investigation of rheological properties, finite element modeling, and in-depth computational and analytical interpretation of results was carried out.

It has been established that the decrease in the rolling temperature from 500 to 400 °C intensifies the slippage of metal in contact with rolls, resulting in the strain kinematic state of the rolled workpiece changing significantly. In particular, the speeds decrease and the configuration of the metal movement trajectories changes.

The axial speed component decreases to the greatest extent, from 66.35 at 500 °C to 44.13 at 400 °C. The circumferential speed, as well as the strain rate intensity associated with it, are significantly less dependent on temperature.

In general, according to the nature of the effect on the strain kinematic state, the temperature reduction acts similar to the decrease in feed angle of about 4°, and, in particular, increases the rolling time, nonuniformity of deformation, tightening, and temperature effect of deformation heating.

## Figures and Tables

**Table 1:** Parameters of model for simulation of RSR process.

Parameter	Value
Workpiece: - diameter, mm - length, mm - workpiece temperature, °C - heat conductivity, W/m·K - heat capacity, J/kg·K	30 150 400, 500 130 960
Rolls: - diameter in pitch point, mm - rotary velocity *n*, rpm - temperature, °C - material	90 60 20 55NiCrMoV7 (EN)
Elongation ratio *µ*	2.0
Friction factor between workpiece and rolls	1.0

**Table 2:** Trajectory and speed parameters of RSR at different temperatures.

Parameter	Rolling Temperature, °C	
	400	500
Axial component of speed on the exit, *V*_x1_ (mm/s)	44.17	66.35
Tangential component of speed on the exit, *V*_τ1_ (mm/s)	140.96	158.84
Speed on the exit *V _β_*_1_ (mm/s)	147.71	172.14
Angle of surface trajectory on the exit, *β*_1_ (degree)	17.4	22.7
Length of axial trajectory, *L_oc_* (mm)	44.3	44.5
Axial component of speed on the entrance, *V*_x0_ (mm/s)	23	33.04
Tangential component of speed on the entrance, *V*_τ0_ (mm/s)	188.39	203.39
Speed on the entrance, *V _β_*_0_ (mm/s)	189.78	206.05
Angle of surface trajectory on the entrance, *β_0_* (degree)	6.96	9.23
Length of surface trajectory in the deformation zone, *L_β_* (mm)	128.40	102.46

## Data Availability

The data presented in this study are available on request from the corresponding author.
